# Efficiency of Iterative Metal Artifact Reduction Algorithm (iMAR) Applied to Brain Volume Perfusion CT in the Follow-up of Patients after Coiling or Clipping of Ruptured Brain Aneurysms

**DOI:** 10.1038/s41598-019-55792-6

**Published:** 2019-12-19

**Authors:** Arsany Hakim, Manuela Pastore-Wapp, Sonja Vulcu, Tomas Dobrocky, Werner J. Z’Graggen, Franca Wagner

**Affiliations:** 1 University Institute of Diagnostic and Interventional Neuroradiology, Bern University Hospital, Inselspital, University of Bern, Bern, Switzerland; 2 Support Center for Advanced Neuroimaging (SCAN), University Institute of Diagnostic and Interventional Neuroradiology, Bern University Hospital, Inselspital, University of Bern, Bern, Switzerland; 3 Department of Neurosurgery, Bern University Hospital, Inselspital, University of Bern, Bern, Switzerland

**Keywords:** Brain imaging, Medical research, Cerebrovascular disorders

## Abstract

Metal artifacts resulting from coiling or clipping of a brain aneurysm degrade image quality and reduce diagnostic usefulness of computed tomography perfusion CTP. Our aim was to assess the diagnostic value of the iterative metal artifact reduction algorithm (iMAR) in CTP studies after coiling or clipping of ruptured intracranial aneurysms. Fifty-eight CTP exams performed in 32 patients were analysed. iMAR was applied to the source images from the CT scanner. Perfusion maps were generated from datasets both with and without iMAR, and both datasets were compared qualitatively and quantitatively. Qualitative analysis included evaluation of intensity of artifacts, image quality, presence of new artifacts, and the reader’s confidence in their diagnosis as well as diagnostic impression. Quantitative analysis included evaluation of tissue attenuation curves, evaluation of region of interest (ROI)-based measurement of perfusion values at levels that do and do not contain metal, compared to previously published reference ranges of perfusion values. Our results showed that application of iMAR reduced artifacts and significantly improved image quality. New artifacts were observed adjacent to metallic implants, but did not limit the evaluation of other regions. After correction for artifact readers’ confidence in their diagnosis increased from 41.3% to 87.9%, and the diagnostic impression changed in 31% of the exams. No difference between tissue attenuation curves was found. For slices without metal, no difference was noted between values measured before and after iMAR, and the total number of ROIs in the reference range of perfusion values was unchanged. At the level of the metal implant, 89.85% of ROIs obtained before using iMAR showed calculation errors. After using iMAR, only 1.7% showed errors. Before iMAR 3.1% of values were in the reference range, whereas after iMAR this increased to 33.1%. In conclusion, our results show that iMAR is an excellent tool for reducing artifacts in CTP. It is therefore recommended for use in clinical practice, particularly when severe artifacts are present, or when hypoperfusion is suspected at the level of the coil or clip. After the application of iMAR, the perfusion values at the level of the metal can be better calculated, but may not lie within the reference range; therefore, quantitative analysis at the level of artifacts is not advisable.

## Introduction

Vasospasm and delayed cerebral ischemia (DCI) remain the most important causes of morbidity and mortality following ruptured brain aneurysms and occur in up to 70% of patients 3–14 days after initial hemorrhage^1^. The diagnosis and treatment of DCI depend on early detection, and computed tomography (CT) imaging plays an important role. CT perfusion (CTP) can detect perfusion changes resulting from vasospasm, including changes at the microcirculation level, and is therefore valuable in these patients^2^. In addition, CTP can differentiate between patients with reversible ischemia and those who will progress to infarctions^3^. In addition to qualitative analysis, quantitative analysis is also useful as it may predict vasospasm even in regions with no visible abnormalities^4^. Combining CTP with CT angiography (CTA) further improves patient outcomes and lowers health care costs^5^. CTP has gained prominence since the introduction of volume perfusion CT with wider coverage on the Z-axis, due to its ability to detect the involvement of different territories, which commonly occurs in vasospasm^6^. However, implanted coils and clips cause significant artifacts that lead to image distortion, degrade image quality, and decrease the ability to detect perfusion changes at the level of artifacts. This limits image evaluation and the value of whole-brain perfusion studies in these patients. Reducing artifacts can improve evaluation and depiction of subtle perfusion changes, which may affect patient management.

Previously, satisfactory methods for reducing these artifacts were lacking. Recently, iterative metal artifact reduction algorithm (iMAR, Siemens Healthcare)^7^, was developed and has shown favourable results in various body regions^8^.

The aim of our study was to evaluate the efficiency of the iMAR algorithm in reducing artifacts resulting from coils or clips in CTP, and to establish whether it is possible to perform qualitative or quantitative analysis of images processed with iMAR in regions containing metal as well those that do not contain metal.

## Materials and Methods

The institutional review board of the University Hospital Bern and the local ethics committee (Kantonale Ethikkomission Bern, Switzerland) approved the access to data for this retrospective study of patients who had undergone CTP after coiling or clipping of brain aneurysms between February and September 2016 at our institute. All patients scanned in this time period were considered for inclusion in the study. This study was conducted in accordance with the guidelines of the Declaration of Helsinki^9^. Written informed consent was obtained from all enrolled patients. Five patients refused to participate, and one patient was under 18 years of age; these 6 patients were excluded from the study. All CT studies were clinically indicated, and no CT scans were performed for research purposes.

### CT acquisition

In accordance with our institute’s protocol, patients with aneurysmal subarachnoid hemorrhage underwent multimodal CT within the first 5 days after aneurysm treatment. CT was repeated as necessary, depending on clinical status. Our standard protocol includes unenhanced CT and CTA followed by a CTP.

All scans were performed on a 128-multidetector CT (SOMATOM definition edge, Siemens Healthcare, Erlangen, Germany) with an adaptive 4D spiral solution that included a toggle table with a shuttle mode technique, which allowed coverage of 9.5 cm of the brain on the Z-axis. Thirty millimeters of intravenous contrast (Imeron 400, Bracco imaging, Konstanz, Germany), followed by a 30 ml saline flush, were administered into a cubital vein at a flow rate of 5 ml/s using a mechanical injector (Swiss Medical Care, Lausanne, Switzerland). The acquisition parameters were as follows: 350 mA; 80 kV; exposure time 570 ms; matrix 512 × 512; FOV 20 cm; spiral pitch factor 0.5; single collimation width 1.2 mm, and kernel H20f. In total, 30 contrast phases were obtained within 46.3 s. The computed tomography dose index (CTDI_vol_) was 223.29 mGy and the dose-length product (DLP) was 2633 mGycm. The scanned volume was reconstructed in 5 mm thick slices, which were used later in the postprocessing. In this study, the gray-scale images obtained from the CT scanner were referred to as source images.

### iMAR

The perfusion source images were processed with iMAR neurocoils, which is an add-on tool in the scanning software. The gray-scale perfusion images required approximately 3 minutes of processing. The images were referred to as “uncorrected” before processing with iMAR and as “corrected” afterwards. Both the corrected and uncorrected datasets were then transferred to PACS for further evaluation and to a multimodality workstation (syngo.via VB10B, Siemens Healthcare, Erlangen, Germany) for post-processing and generation of perfusion maps.

### Perfusion post-processing

**Figure 1:**
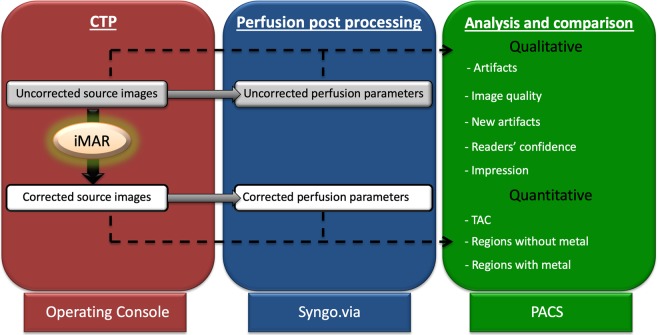
Flowchart showing the study workflow. The figure shows the steps performed in operating console (red), the postprocessing of perfusion maps and quantitative analysis (blue) and the image evaluation in PACS workstation (green).

Semi-automatic post-processing was performed while a neuroradiologist checked the accuracy of each of the following steps: (1) motion correction, (2) noise reduction, (3) bone subtraction using anatomical information from the baseline phase, (4) vessel segmentation (the artery and vein were automatically detected), and calculation of the arterial input function (AIF). The automatic vessel segmentation performed by syngo.via resulted in identical post-processing of both datasets without user interference, allowing an unbiased comparison following post-processing. Finally, time to peak (TTP) was calculated using maximum slope, and cerebral blood flow (CBF), cerebral blood volume (CBV), mean transit time (MTT), time to drain (TTD) and time to maximum (T_max)_ maps were calculated using deconvolution. These perfusion maps were saved as red/green/blue (RGB)-coloured scale images and sent to the PACS (Fig. 1).

**Figure 2:**
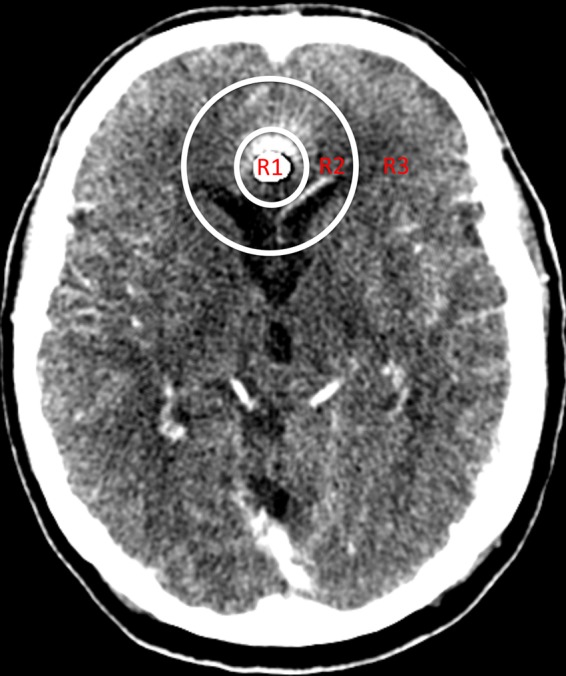
Source image from CTP after reconstruction with iMAR from a 74-year-old male after coiling of a ruptured aneurysm of the pericallosal artery. The 3 regions used for the evaluation are illustrated as R1–R3: R1: inside the 2 cm diameter circle; R2: the region between the 2 cm and 5 cm diameter circles; and R3: the region outside the 5 cm diameter circle with the coil packet in its centre.

Tissue attenuation curves (TACs) were documented for each scan for both the corrected and uncorrected datasets. A ROI was drawn in a reference region (RR) in a slice that did not contain metal, and 3 other ROIs (R1–R3) were drawn at the level of maximum artifacts as follows: (R1) inside a 2 cm diameter circle, (R2) between the 2 cm and a 5 cm diameter circle, and R3) outside the 5 cm diameter circle with the implanted coil or clip at its centre (Fig. 2). The same ROI localization was chosen for both corrected and uncorrected datasets. Visible hypoperfused regions were avoided or excluded, so that normal values of our patients could be compared with published reference standards^10^. Regions affected by foreign bodies, other than coils or clips were avoided. The values of the perfusion parameters (CBV, CBF, MTT, TTD, and TTP) were saved in a table format separately for each of the corrected and uncorrected datasets for evaluation and comparison.

### Evaluation

Image analysis was performed on a certified reporting workstation (Sectra IDS7, Linköping, Sweden) by 2 independent board-certified neuroradiologists (A.H. and F.W.). Any disagreement was resolved by consensus. A blinded analysis of the corrected and uncorrected datasets was not realistic because artifact reduction was obvious to the readers after processing using iMAR. The readers were blinded to the clinical course, angiographic findings, final imaging findings, and patient outcomes.

#### Qualitative assessment

Artifact evaluation. The artifact evaluation was performed on the source images at the level of the coil or clip. The same slice was selected for both, the corrected and uncorrected datasets. A 4-point Likert scale (Table 1) was used in the 3 regions (R1–R3)^8^. An RR was selected from another slice that did not contain metal and was rated using the same 4-point Likert scale.

**Table 1:** 4-point Likert scale scores used in the evaluation of the artifacts and image quality.

Score	Artifacts (source image)	Image quality (perfusion maps)
0	Totally obscured: Anatomy is not visible	Worst: very poor
1	Severe: Strong artifacts and limited evaluation of anatomical structures	Poor
2	Moderate: Artifacts present, but assessment of anatomical structures is not impaired	Fair
3	None to very mild	Best: good to excellent

Image quality. The same 4 regions (R1–R3 and RR) were also evaluated for image quality on perfusion parameters using a 4-point Likert scale (Table 1).

New artifacts. The corrected dataset was checked for new artifacts.

Readers’ confidence. The readers’ confidence in interpreting the images (corrected and uncorrected) was noted. This confidence level was based on the overall impression of the image, including its quality and the presence of artifacts.

Impression. Potential changes in diagnosis after using iMAR were evaluated by assessing a selected slice with artifacts in both the corrected and uncorrected datasets and recording the reader’s impression: no perfusion abnormality, hemodynamic hypoperfusion (increased MTT), reversible ischemia (increased MTT and decreased CBF), or irreversible ischemia (increased MTT; decreased CBF and decreased CBV)^11^. The impressions recorded for the corrected and uncorrected perfusion maps were compared to evaluate potential changes in diagnosis after artifact reduction.

#### Quantitative assessment

**Figure 3:**
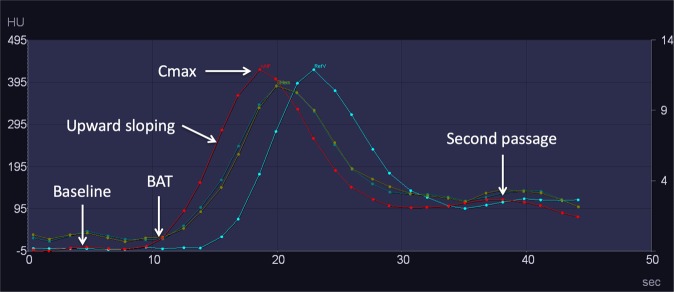
Tissue attenuation curves obtained from a CTP study processed with iMAR from a 58-year-old female after coiling of a ruptured left posterior communicating artery aneurysm. The arterial input function is represented by the red curve, the venous reference by the blue curve, and the tissue curves (left and right hemispheres), by the green curves. While comparing the curves from the corrected and uncorrected datasets, special attention was paid to the marked points (baseline, bolus arrival time (BAT), upward sloping, contrast maximum (C_max_), and second passage peak).

First step. The aim of the first step was to evaluate whether iMAR affected the procedure of perfusion post-processing. It was performed by visually comparing TAC morphologies of corrected and uncorrected datasets. Special attention was paid to the characteristic features of the curve^12^, i.e., baseline, bolus arrival time, upward sloping, C_max_, downward sloping and the second passage peak (Fig. 3).

Second step. This step aimed to determine whether iMAR affects perfusion values at levels that do not contain metal artifacts. This involved comparing the values obtained from RRs in both corrected and uncorrected datasets and comparing both these values with published reference values for normal brain tissues published by Abels *et al*.^10^.These reference values were chosen because, as in our study, these values had been obtained using a Siemens’ scanner and post-processing equipment.

Third step. The aim of this step was to find out whether perfusion values at the level of artifacts could be obtained after application of iMAR and whether these values were plausible. This was done by comparing the values obtained from ROIs (R1–R3) in both the corrected and uncorrected datasets with the previously mentioned published reference values to determine which values lay within the reference range^10^. The presence of even one perfusion parameter value outside the reference range was considered as being incorrect for the entire ROI.

From the data collected, values from each perfusion parameter were compared with reference values to assess whether a particular perfusion parameter was more accurate than the others in regions with artifacts. The aims were (a) to find out which parameter was more resistant to artifacts, with the idea that this parameter could be used if a quantitative analysis is required at the level of artifacts, and (b) to determine whether there was an improvement in these parameters after iMAR application.

### Statistics

The statistical analyses were performed using IBM SPSS Statistics for Windows Version 22.0. All statistical tests were 2-sided; a *p*-value < 0.05 was considered statistically significant. The Wilcoxon signed-rank test was used to compare the ratings of the uncorrected and corrected datasets. The inter-rater reliability was calculated according to Cohen’s kappa. Because of the small sample size of patients who had undergone clipping compared to patients who had undergone coiling, and the non-normally distributed variables within these groups, the non-parametric Mann-Whitney U test was applied to compare the ratings of these 2 groups. Paired t-tests were used to compare values between the corrected and uncorrected parameters at levels that did not contain metal artifacts. Pearson’s chi-square test was used to compare the total perfusion values within the reference range and the total perfusion values that could not be calculated by the software, for the corrected and uncorrected datasets.

## Results

### Patient characteristics

This study included 32 patients: 10 males (mean age 57.2, range 52–73 years) and 22 females (mean age 62.4, range 40–79 years). Thirty of the 32 patients were examined during the vasospasm phase and underwent up to 5 CTP studies according to clinical indications and depending on the clinical course. Fourteen of these patients did not develop clinical or angiographic evidence of vasospasm, whereas 16 patients were diagnosed with vasospasm/DCI. The diagnostic criterion for vasospasm was evidence of arterial narrowing compared with the parent vessel or baseline exam^1^. DCI was defined as an acute neurological deficit fulfilling the following criteria: a decrease in Glasgow Coma Scale score of at least 2 points and/or an increase in NIHSS of at least 2 points. Of these patients with vasospasm, 11 were treated with triple H (3H; hypertension, hypervolaemia and haemodilution) therapy, and 5 were treated with both 3H therapy and intraarterial nimodipine. The other 2 patients included in the study had been previously treated for aneurysms and underwent CTP during a stroke work-up due to acute neurological symptoms. Altogether, 58 CTPs performed in 32 patients were evaluated.

### Aneurysm location and foreign bodies

Four patients had an aneurysm in the posterior circulation and the others had aneurysms in the anterior circulation. Overall, 26 patients underwent endovascular coil embolization, and 6 underwent surgical clipping. The foreign materials examined consisted of platinum-tungsten alloy (Target coils), platinum alloy (Deltamaxx coils), titanium alloy (Sugita clips) and titanium and titanium alloy (Lazic clips). In 2 patients, the clip model was unknown.

The scanned regions contained other metallic objects, such as bolt-connected external ventricular drains (EVDs), intracranial pressure monitoring probes (ICP-probes), CranioFix clamps and skin clips. Due to the wide variation in sizes and types, the effect of iMAR on these materials was not evaluated. In 28 of the 58 scans, an orbit shield was included in the caudal part of the scan coverage.

#### Qualitative assessment

**Figure 4:**
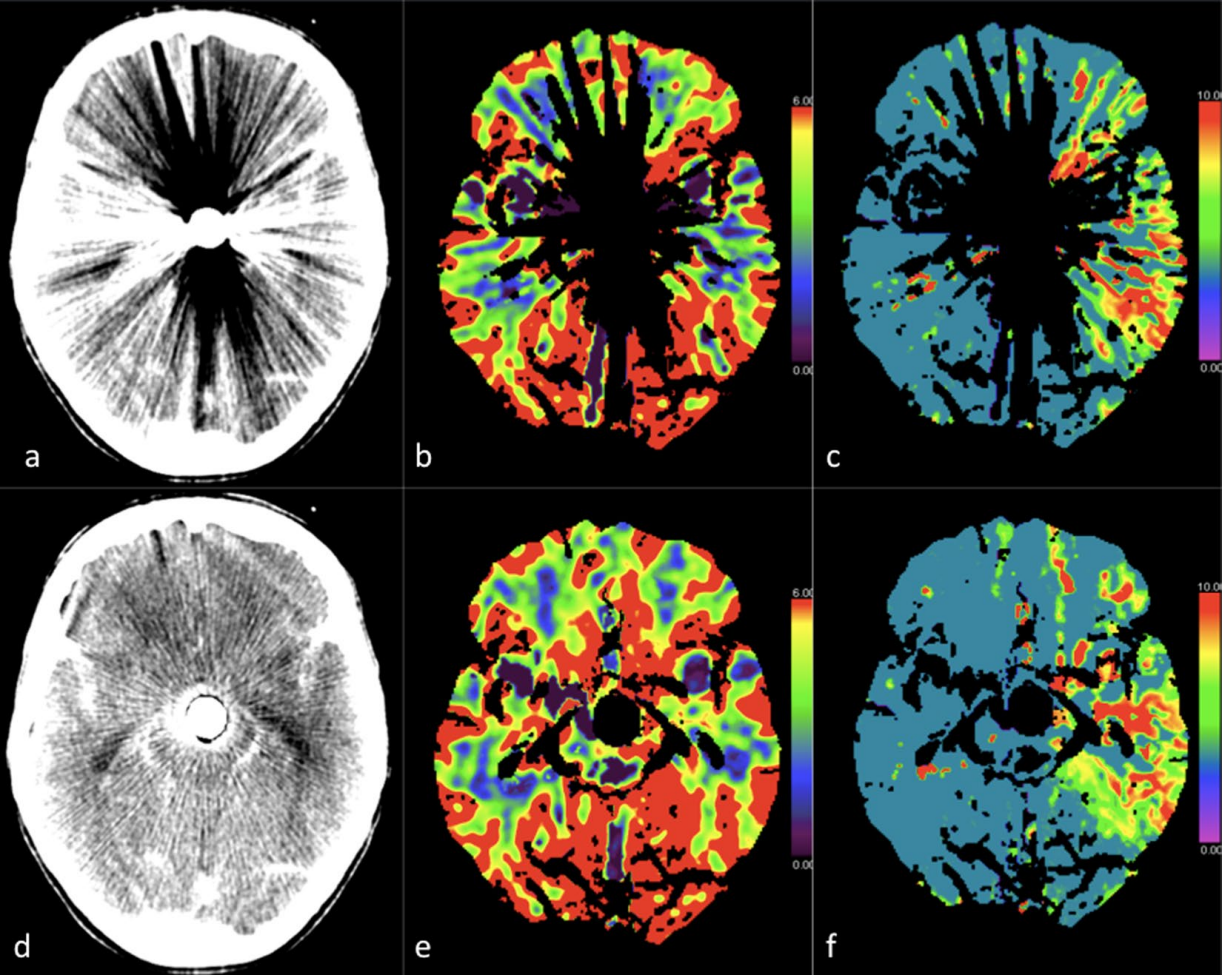
CT perfusion study of a 60-year-old female patient after coiling of a ruptured basilar tip aneurysm. The upper panel (a–c) show images before, and the lower panel (d–f) show images after iMAR application. Obvious metal artifact reduction was apparent after iMAR application, which improved the detection of MTT prolongation in the left temporal lobe (**c**,**f**) and increased rater confidence. Rater confidence in analysing the CBV (**b**,**e**), which showed no asymmetry even after artifact reduction, was also increased. Note: A hypodense ring artifact surrounded by a hyperdense ring was observed around the metal in the source images processed with iMAR (**d**).

Artifact evaluation. The uncorrected datasets displayed extensive artifacts obscuring anatomy in all the 3 regions evaluated as follows: 98.2% in R1, 93.1% in R2, and 51% in R3 (Fig. 4).

In the corrected datasets, artifact reduction was observed as follows: a 36.2% reduction in R1, a 94.8% reduction in R2 and a 79.3% reduction in R3 (see Supplementary Fig. S1). Only one scan (1.75%) in R3 showed a worsened artifact score (from moderate to poor). In this scan, an orbit shield was present at the coil level. An almost perfect agreement was observed between the ratings of the 2 readers across all 3 regions, k > 0.801, *p* < 0.001 (disagreement occurred on 6/116 ROIs in R1, 11 ROIs in R2, and 16 ROI in R3).

In R1, artifact reduction in patients with clips (mean rank 40.38) was significantly better than that in patients with coils (mean rank 27.76); (U = 113, *p* = 0.021). In R2, artifact reduction was not significantly different between patients with coils (mean rank 30.26) and those with clips (mean rank 24.75); (U = 162, *p* = 0.35). Similarly, in R3, artifact reduction was not significantly different between patients with coils (mean rank 30.95) and with clips (mean rank 20.44) (U = 127.5, *p* = 0.089) (see Supplementary Fig. S2).

No differences in scores were observed between the datasets in the RR.

Image quality. The image quality in the uncorrected dataset was degraded in all 3 of the regions evaluated. The corrected dataset showed improved image quality as follows: 13.8% improvement of the scans in R1, 89.6% improvement in R2, and 63.8% improvement in R3 (Fig. 4).

Reduced image quality was observed in 3.4% of the scans in R2 and in 5.1% of the scans in R3 (see Supplementary Fig. S3). All affected regions were identified in 3 different exams in which an orbit shield was present at the coil level, and in one exam, additional motion artifacts were present.

An almost perfect agreement was observed between the ratings of the 2 readers for image quality in regions R1 and R2, k > 0.839, *p* < 0.001. In R3, the agreement was substantial, k = 0.600, p < 0.001 (disagreement occurred on 2/116 ROIs in R1, 9 ROIs in R2 and 22 ROIs in R3).

In R1, the image quality was significantly improved after iMAR in patients with clips (mean rank 40.06; U = 115.5, *p* = 0.001) compared with that in patients with coils (mean rank 27.81). In R2, the improvement was significantly better in patients with coils (mean rank 31.93) than in patients with clips (mean rank 14.31; U = 78.5, *p* = 0.001). Similarly, in R3, the improvement was significantly better in patients with coils (mean rank 31.54) than in those with clips (mean rank 16.75 U = 98, *p* = 0.015) (see Supplementary Fig. S4).

No differences in scores were observed between the datasets in the RR.

New artifacts. In 57/58 exams (98.2%), additional artifacts were observed in region R1, namely a small hypodense rim around coils or clips surrounded by a hyperdense rim. These artifacts were confined to R1 and did not limit the evaluation of other regions (Fig. 4d). In 28 exams where an orbit shield was present a considerable number of artifacts were observed in both source images and perfusion maps, and these artifacts were aggravated after reconstruction with iMAR. However, other than those described above, no new artifacts were detected after application of iMAR.

Readers’ confidence. When assessing the uncorrected dataset, the readers were not confident in their interpretations in 34/58 exams (58.6%). For the corrected dataset, the readers changed their evaluation to confident in 28 of these 34 exams (82.3%). In 28/58 exams (48.2%), the readers’ confidence was unchanged after correction. In only 2/58 exams (3.4%) were the readers not confident after iMAR application, even though they had been confident about the uncorrected datasets. Overall, the readers’ confidence increased from 41.3% to 87.9% after iMAR application.

Impression. In 18 exams (31%), the impressions of the readers regarding the selected slice changed after iMAR application. However, this change did not affect the overall impression of the examination due to the presence of perfusion changes in other slices from these patients.

#### Quantitative assessment

First step: effect of iMAR on the post-processing of CTP data. Data processed with iMAR showed no differences in post-processing steps all of which were performed smoothly without generating any error messages. In all corrected datasets, curve morphology for AIF, venous reference (RefV), and right and left hemispheric curves showed regular patterns.

In 56 of the 58 exams (96.5%), the TAC-morphology showed no difference between corrected and uncorrected datasets. In 2 exams (3.4%) obtained from a single patient, slight changes in TAC were seen: in the first exam, the peak of the RefV was slightly wider after the application of iMAR; and in the second exam, the peak was slightly narrower after the application of iMAR. In both these exams, an orbit shield was included in the scan field.

Second step: effect of iMAR on perfusion values at levels distant from artifacts. The values from RR were calculable without errors for both datasets. The results of paired t-tests comparing the values of perfusion parameters between the corrected and uncorrected datasets were non-significant for all parameters: CBF (t = 0.782, *p* = 0.438), CBV (t = 1.668, *p* = 0.101), MTT (t = 0.757, *p* = 0.452), TTD (t = 0.514, *p* = 0.609), and TTP (t = 1.963, *p* = 0.054). Each dataset included 51 values (87.9%) within the previously mentioned reference range and 7 values (12.1%) outside it.

Third step: the Possibility of obtaining perfusion values at the level of artifacts and whether these values are plausible. R1. Only one exam (1.7%) yielded uncorrected dataset values that were inside the reference range, one exam (1.7%) yielded values outside the reference range, and one exam had to be excluded due to visible hypoperfusion in this region. The values from the remaining 55 exams (94.8%) displayed an error message in calculation by the post-processing software due to extensive artifacts. After the application of iMAR, the values from 54 of those 55 exams (98.1%) could be calculated without any error message. The values from only 7 of those scans (12.9%) were inside the reference range whereas the values from 38 exams (70.3%) were outside the reference range. The values from 9 exams were excluded due to an impractical location (i.e., there was not enough brain tissue around the coil to allow a ROI to be precisely drawn). The exam that yielded values within the reference range before iMAR remained within the reference range, and the exam that yielded values outside the reference range before iMAR remained outside the reference range (see Supplementary Fig. S5a). The statistical analyses comparing the number of ROIs with values that were not calculated by the software showed a significant difference between the uncorrected and corrected datasets (chi-square = 100.7, *p* < 0.001) and statistically significant result for the comparison of the ROIs within the reference range (chi-square = 5.9, *p* = 0.015).

R2. Only one exam (1.7%) yielded values that were inside the reference range before iMAR, and these values remained inside the reference range after the application of iMAR. The values from 7 exams (12%) were outside the reference range in the uncorrected datasets. Four of them (57.1%) showed values inside the reference range after the application of iMAR, whereas the values for the other 3 (42.8%) remained outside the reference range. The values of the other 50 exams (86.2%) displayed an error message in their calculation by the post-processing software. After iMAR it was possible to calculate values for 49 of those 50 exams (98%): 18 of them (36.7%) were inside the reference range, 29 (59.1%) were outside it, and 2 were excluded due to hypoperfusion (see Supplementary Fig. S5b).

The statistical analysis showed a significant difference comparing the uncorrected and corrected datasets (chi-square = 84.0169, *p* < 0.001) for the ROIs that could not be calculated by the software, and a significant difference for the comparison of the ROIs within the reference range (chi-square = 25.43, *p* < 0.001).

R3. The values from 3 exams (5.1%) were within the reference range before iMAR. After iMAR, 1 of these values remained within the reference range (33.3%), whereas the other 2 (66.6%) were outside the reference range. The values from 21 of the 58 exams (36.2%) before iMAR were outside the reference range. After application of iMAR, 13 of those 21 exams (61.9%) remained outside the reference range, but the values from 8 exams (38%) were inside the reference range. In the uncorrected datasets, the values from the remaining 34 exams (58.6%) displayed an error message during their calculation by the software. After iMAR, values for 33 of those 34 exams (97%) could be calculated: 18 (54.5%) were inside the reference range and 13 (39.3%) were outside the reference range. The values from 2 exams were excluded (see Supplementary Fig. S5c).

There was a statistically significant difference between the values that could not be calculated before and after the application of iMAR (chi-square = 44.56, *p* < 0.001) and the comparison of the ROIs within the reference range was also significant (chi-square = 17.52, *p* = 0.001).

Overall, for the 3 regions together, 139 of 174 ROIs (89.85%) could not be calculated before using iMAR, but after iMAR, only 3 of the 174 (1.7%) were still not calculable. The total number of ROIs with values within the reference range before iMAR was 5 of 160 (3.1%), whereas after iMAR it was 53 of 160 (33.1%).

The frequency over all 3 regions was also significantly different in the comparison of the uncorrected and corrected datasets for the ROIs that could not be calculated by the software (chi-square = 220.03, *p* < 0.001) and for the comparison of the ROIs within the reference range (chi-square = 47.66, *p* < 0.001).

In both corrected and uncorrected datasets, the most accurate perfusion parameter in the regions of the artifacts was the TTP, followed by MTT and CBF. The statistical analysis of these parameters showed no significant difference in the uncorrected dataset (chi-square = 8.7, *p* = 0.07), but there was a significant difference between them in the corrected dataset (chi-square = 66.62, *p* < 0.001) (see Supplementary Fig. S6).

## Discussion

We assessed the ability of iMAR to reduce artifacts resulting from coils and clips used to treat brain aneurysms in CTP studies.

Our results can be summarized as follows: (1) after the application of iMAR, the process of perfusion calculation remains the same, and there is no significant change in the TACs; (2) the quality of CT slices that did not contain metal is not affected after iMAR application and values in these regions remain within the normal reference range^10^ as in the unprocessed images; (3) iMAR significantly reduces artifacts in images that include coils or clips, especially in regions not directly adjacent to the implanted materials and there was a significant increase in the number of ROIs with calculable perfusion values after iMAR that were not calculable beforehand due to extensive artifacts; however, the values obtained do not always lie within the reference values published for normal tissue perfusion; (4) iMAR application noticeably improves the image quality of perfusion maps, which increases diagnostic confidence and the visibility of perfusion changes; and (5) images processed with iMAR may reveal findings that were obscured in the original datasets due to the presence of artifacts.

Metallic artifacts typically obscure normal anatomy, mask potential pathological findings, and decrease the value of volume CTP, which plays an important role in the follow-up of patients with vasospasm and DCI. Reducing such artifacts is critical to enable better interpretation of perfusion images in these patients. Therefore, iMAR is a highly valuable, fast and simple add-on tool that can significantly improve image quality.

iMAR is projection-based metal artifact reduction algorithm, which approaches photon starvation and is based on previously developed algorithms, namely frequency split metal artifact reduction (FSMAR) and normalized metal artifact reduction (NMAR)^13–15^. iMAR segments the corrupted projection data, then replaces them with estimated corrected values. In contrary to other approached of metal reduction, iMAR compensates for photon starvation without increasing of the radiation dose given to the patient and without reducing the iodine contrast-to-noise-ratio. Another advantages of iMAR is that the algorithm can be applied retrospectively, so that the radiologist can decide if such approach is needed after viewing the original images^16^.

Other approaches to photon starvation include simple techniques such as changing the peak voltage and tube current. This approach is only suitable for minor artifacts and increases the radiation dose to the patient. The use of a dual energy scanner (with single or dual source) is an approach to overcome beam hardening. Through the acquisition of 2 energy spectra from the same tissue, a virtual monochromatic image can be synthesized, which is able to reduce the effect of beam hardening if acquired at high energy; accordingly, there will be less tissue contrast with such high energy levels. Another drawback of the dual energy technique is that it cannot be performed retrospectively, so that optimal energy needs to be decided before image acquisition^16^. The dual energy technique is usually insufficient to reduce artifacts caused by photon starvation due to large metal size or high atomic number^16^.

More recently, advanced machine learning approaches have been used to reduce artifacts. Examples include deep learning based sinogram correction using convolutional neural network (CNN)^17^, fusion of the information from the original and corrected images to suppress artifacts using CNN^18^, or training an adversarial deep network to fill in the missing data from artifacts, directly in the projection domain^19^. But to the best of our knowledge none of these approaches were tested with perfusion images.

Previous studies have shown the efficiency of iMAR in different body regions^8,20^. Other studies have evaluated the performance of iMAR in non-contrast CT and CTA during follow-up after coiling or clipping^21–25^. The results of these studies indicate that iMAR significantly reduces metal artifacts^26^, increases overall diagnostic quality^21^, allows better assessment^23^ and increases vessel segment visualization^24,25^. The findings of our study are consistent with the current literature and all together confirm that iMAR is valuable for improving the quality of images obtained by 3 different techniques used to monitor patients with subarachnoid haemorrhage, vasospasm and DCI (CT, CTA and CTP).

However, the images reconstructed with iMAR were not artifact-free, particularly in narrow regions directly adjacent to metallic implants. A hypodense area around metallic objects (Fig. 4d) was a frequently observed artifact. This was due to loss of information in the interpolation process, which could not be recovered during iteration. Similar findings have also been reported in other studies^23^. Such artifacts may impair evaluation of neighbouring vessels^22^ but did not seem to affect our results.

Although qualitative assessment found obvious improvements were observed in other regions outside the 2-cm circle, a few exams showed no improvement, and an even smaller number showed artifact worsening. In our data analysis, decreased image quality and increased artifacts were observed in 6 instances in 3 different exams. In 4 of these instances (in 2 different exams) artifacts were due to presence of an orbit shield^27^ at the coil level, and in the 2 other instances (in one exam) an orbit shield was present at coil level as well as additional motion artifacts. Therefore, other factors may play a role in decreasing the quality of the data. In our workflow, applying iMAR after performing motion correction was not technically possible.

In the quantitative assessment the values for almost all of the patients could be calculated after iMAR. This represents a step forward in artifact reduction in the complex procedure of perfusion calculation.

Previous studies have attempted to test the accuracy of Hounsfield units (HUs) in the structures adjacent to metallic foreign bodies in the white matter or by using a phantom^13,22,28^. Axente *et al*. found residual errors in CT numbers as well as errors induced by the correction algorithm^13^. The results of our quantitative assessment reflect such errors, as the perfusion values were not always within the reference range. Such errors in HU calculation may not be the only reason for false perfusion values, as orbit shield and motion artifacts were frequently found in the scans showing values that were not within the reference range. This finding was also confirmed in the 2 exams with slightly changed curve morphologies, as in both cases an orbit shield was present in the scan region^27^.

Bier *et al*. reported that iMAR may induce novel artifacts in fine structures adjacent to the metal, such as vessels^22^; thus, caution in the interpretation of the results was advised, as false-positive findings are possible. In perfusion imaging, we also advise obtaining perfusion values in slices that do not contain metal. If perfusion values at the level of artifacts are clinically important, these values should be interpreted with caution. In this situation, the TTP may be the most accurate perfusion parameter, as it showed the best results when compared to other perfusion parameters. In our study, the TTP was calculated using maximum slope. This could explain the better results, i.e., maximum slope performs better than deconvolution in the post-processing of CTP in the presence of artifacts.

The performance of iMAR may differ depending on the type of metal, because higher-density materials with higher atomic number usually result in more artifacts than lower-density materials^29^. Thus clips (atomic number 22) cause less artifacts in CT than coils (atomic number 78)^16^. The size of metal plays also an important role, as photon starvation becomes more pronounced, the bigger the object^16^. Artifacts may be further increased by other materials, such as orbit shields^27^.

The degree of image quality improvement of the perfusion maps may not be the same as the degree of artifact reduction in source images due to post-processing techniques that include steps such as noise reduction and motion correction.

Based on our results, we recommend iMAR application in patients with strong artifacts, but advise that iMAR should be used cautiously where other objects are present (e.g. orbit shields, bolt-connected EVDs, …), or if there are strong motion artifacts. Although the quality improvement in the slices that showed artifacts did not change the final diagnosis in our patient sample, but there is the possibility that in certain cases the perfusion changes could only be detected nearby the clots, which could be at the same level as the artifacts. Such changes in the grey matter imply reaction of the distal vessels^30^ (microvascular circulation). In such cases perfusion imaging will be of the utmost important, as CTA may show no evidence of spasm of the large and middle-sized vessels.

This study has certain limitations. First, we used a retrospective design. Furthermore, the number of patients with clips was insufficient to evaluate the effect of iMAR on different metals. The sizes of the coils and clips varied and an accurate analysis of iMAR performance according to the sizes of the metallic objects was therefore not possible. Other surgical foreign materials, such as CranioFix clamps and bolt-connected EVDs, were not included in our evaluation as this was beyond the scope of the present study and due to the variable usage of these materials according to the clinical situation and timing of the scans. Moreover, although the exact localization of the ROIs’ positions between the 2 datasets was attempted, the distorted anatomy sometimes made it impossible to differentiate between the grey and white matter in the ROI localization. Therefore, the values published by Abels *et al*. from both the grey and white matter were used as a collective range^10^.

## Conclusion

iMAR is an effective tool for improving image quality and reducing artifacts resulting from coils or clips in CTP studies. It does not affect the process of post-processing analysis of whole-brain perfusion or the values of tissue perfusion at the levels that do not contain metal. At the level of artifacts, iMAR improves the calculation of perfusion values. However, these values may not lie within reference range and quantitative analysis at the level of the coil or clip should be avoided. If perfusion values at the level of artifacts are of clinical interest, TTP appears to be the most accurate parameter. iMAR increases readers’ confidence and may uncover relevant findings. Therefore, iMAR is particularly useful in patients with relevant artifacts or suspected perfusion changes at the level of the coil or clip. iMAR should be used cautiously if other foreign bodies are within the scan range or when strong motion artifacts are present.

## Supplementary information

Supplementary material

## Data Availability

The datasets generated during the current study are available from the corresponding author on reasonable request.
