# Supplementary material for: Efficiency of Iterative Metal Artifact Reduction Algorithm (iMAR) Applied to Brain Volume Perfusion CT in the Follow-up of Patients after Coiling or Clipping of Ruptured Brain Aneurysms
Source: Sci Rep. 2019 Dec 19;9:19423. doi: 10.1038/s41598-019-55792-6 (PMC6923436; doi:10.1038/s41598-019-55792-6)
Supplement: Supplementary file 1 — Supplementary material [file 41598_2019_55792_MOESM1_ESM.pdf]

Efficiency of Iterative Metal Artifact Reduction Algorithm (iMAR) Applied to Brain Volume Perfusion CT in the Follow-up of Patients after Coiling or Clipping of Ruptured Brain Aneurysms

Arsany Hakim*, M.D.,[1] Manuela Pastore-Wapp,Ph.D., [2] Sonja Vulcu, M.D. [3] Tomas Dobrocky, M.D.,[1] Werner J Z'Graggen, M.D.[3] and Franca Wagner, M.D.[1]

Supplementary material

P	Age	Sex	Aneurysm location	Aneurysm size (DSA)	Aneurysm treatment	No. of coils/clip	Model/material	Vasospasm	Therapy for vasospasm	No. of CTP	Notes
P1	73	M	Right ACA	9x8x7 mm	Coiling	11	Target	No	--	1	
P2	60	F	Basilar tip	14.5 x 13 x 16 mm	Coiling	14	Deltamaxx, Target	Yes	3H	2	
P3	51	F	Basilar tip	5.3 x 4.5 x 6.5 mm	Coiling	7	Target	No	--	1	
P4	71	M	Acom	13 x 7 x 8 mm	Clipping	2	Sugita clips	Yes	3H	1	
P5	61	M	Right MCA bifurcation	5 x 6 x 5 mm	Clipping	1	Lazic	Yes	3H	3	
P6	68	F	Acom	N/A	Clipping	1	N/A	N/A	N/A	1	Stroke work-up
P7	56	F	Acom	8 x 5 x 5 mm	Coiling	10	Target	Yes	3H	3	
P8	50	F	Left MCA bifurcation	11 x 6.5 x 11 mm	Clipping	1	Lazic	No	--	1	
P9	55	F	Right Pcom	8 x 4 x 3.5 mm	Coiling	8	Target	No	--	1	
P10	58	F	Right Pcom	7 x 4.4 x 4 mm	Coiling	4	Target	Yes	3H	2	

P11	57	F	Acom	7 x 5 x 4 mm	Coiling	5	Target	No	--	1	
P12	52	M	Right ACA	6 x 5 x 4	Coiling	10	Target	No	--	1	
P13	51	F	Acom	7 x 9 x 6 mm	Coiling	8	Target	Yes	3H & Intraarterial nimodipine	5	
P14	75	M	Acom	5 x 4 x 5 mm	Coiling	4	Target	No	--	2	
P15	72	F	Acom	5 x 2.5 x 2 mm	Coiling	6	Target	Yes	3H & Intraarterial nimodipine	3	
P16	59	M	Left PICA	N/A	Coiling	4	Target	Yes	3H	3	Due to dissection, measurement of size was not possible
P17	69	F	Left Pcom	N/A	Clipping	1	N/A	N/A	N/A	1	Stroke work-up
P18	70	F	Acom	2 x 3 x 2	Coiling	2	Target	Yes	3H and intraarterial nimodipine	3	
P19	61	F	Right MCA	7 x 5 x 5	Coiling	8	Target	No	--	1	
P20	73	F	Acom	16 x 10 x 14	Coiling	7	Deltamaxx & target	No	--	1	
P21	79	F	Acom	6 x 4 x 4	Coiling	4	Target	Yes	3H	4	
P22	59	M	Acom	6 x 8 x 7	Coiling	5	Target	No	--	2	
P23	57	F	Left Pcom	7 x 7 x 5	Coiling	6	Target	Yes	3H	1	
P24	65	F	Right MCA	6 x 4 x 2.5	Clipping	1	Lazic	No	--	1	
P25	70	F	Right ACA	3 x 3 x 3	Coiling	4	Target	No	--	2	
P26	60	M	Acom	8 x 4 x 5	Coiling	4	Target	Yes	3H & Intraarterial nimodipine	1	
P27	57	M	Acom	6 x 9 x 6	Coiling	6	Target	Yes	3H	1	
P28	61	F	Left distal ICA	9 x 7 x 7	Coiling	4	Target	Yes	3H	1	
P29	40	F	Basilar tip	2.3 x 4 x 3.7	Coiling	3	Target	No	--	1	

P30	74	F	Right Pcom	6 x 5 x 6	Coiling	6	Target	Yes	3H	5	
P31	77	F	Acom	3x3x2.5	Coiling	3	Target	No	--	1	
P32	64	M	Acom	10x12x7.5	Coiling	8	Target	Yes	3H & intraarterial nimodipine	1	

Table 1: Clinical data and foreign materials
CTP: CT perfusion, ACA: anterior cerebral artery, 3H: triple H therapy, Acom: anterior communicating artery, MCA: middle cerebral artery, Pcom: posterior communicating artery, ICA: internal carotid artery, PICA: posterior inferior cerebellar artery, N/A: non applicable.

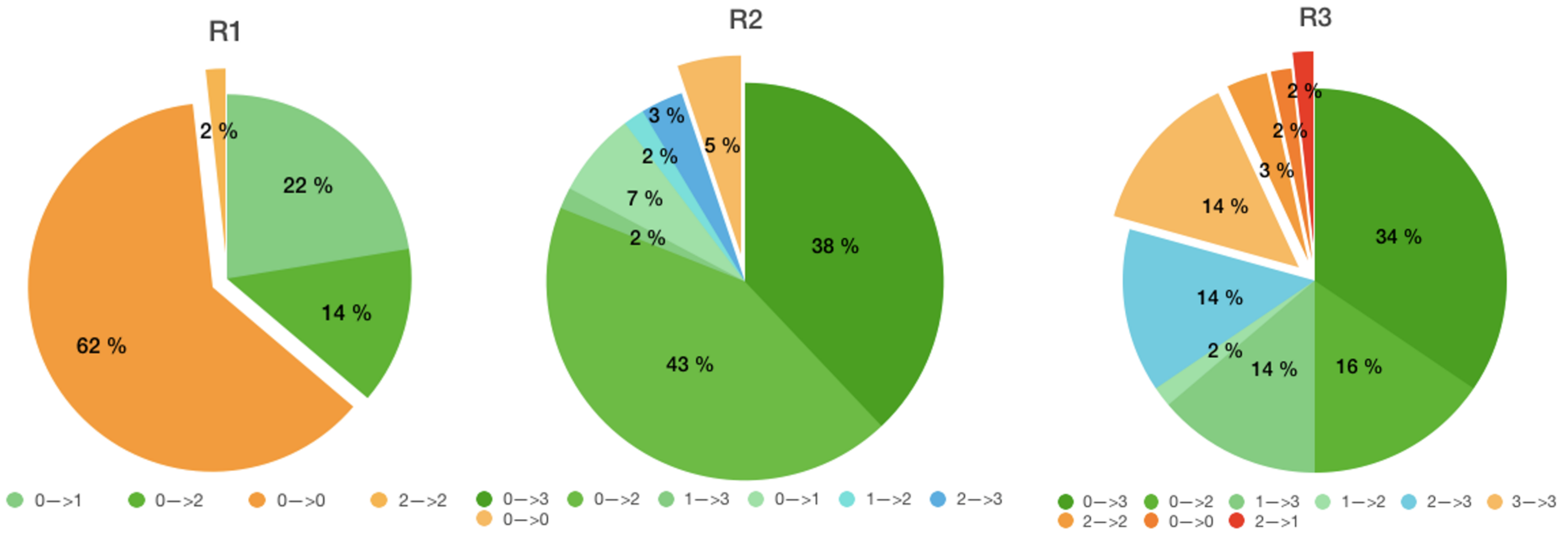

Fig S1: Pie chart of the 3 analysed regions (R1-R3), showing the changes in artifact ratings after application of iMAR (Table 1). The green and blue colours represent reductions in artifact ratings after iMAR application, the orange colour represents unchanged artifact ratings and the red color represents increased artifact ratings after iMAR application. The central tendency differs significantly between the groups (corrected and uncorrected), R1: z=4.16, $p<0.001$, R2: z=6.59, $p<0.001$, R3: z=6.01, $p<0.001$.

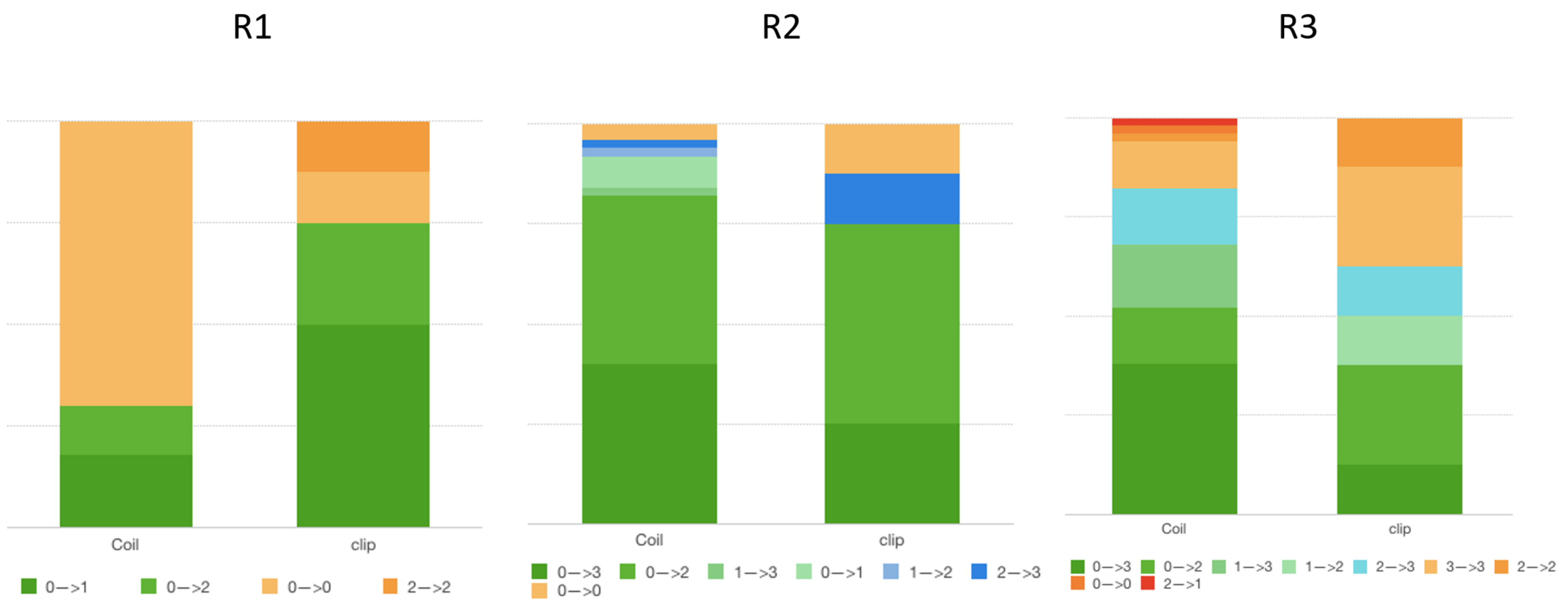

Fig S2: Stacked column chart comparing artifact reduction between patients with clips and those with coils in the 3 analysed regions. In R1, the improvement was significantly better in patients with clips, and in R2 and R3, the improvement was better in patients with coils. But the difference was not statistically significant.

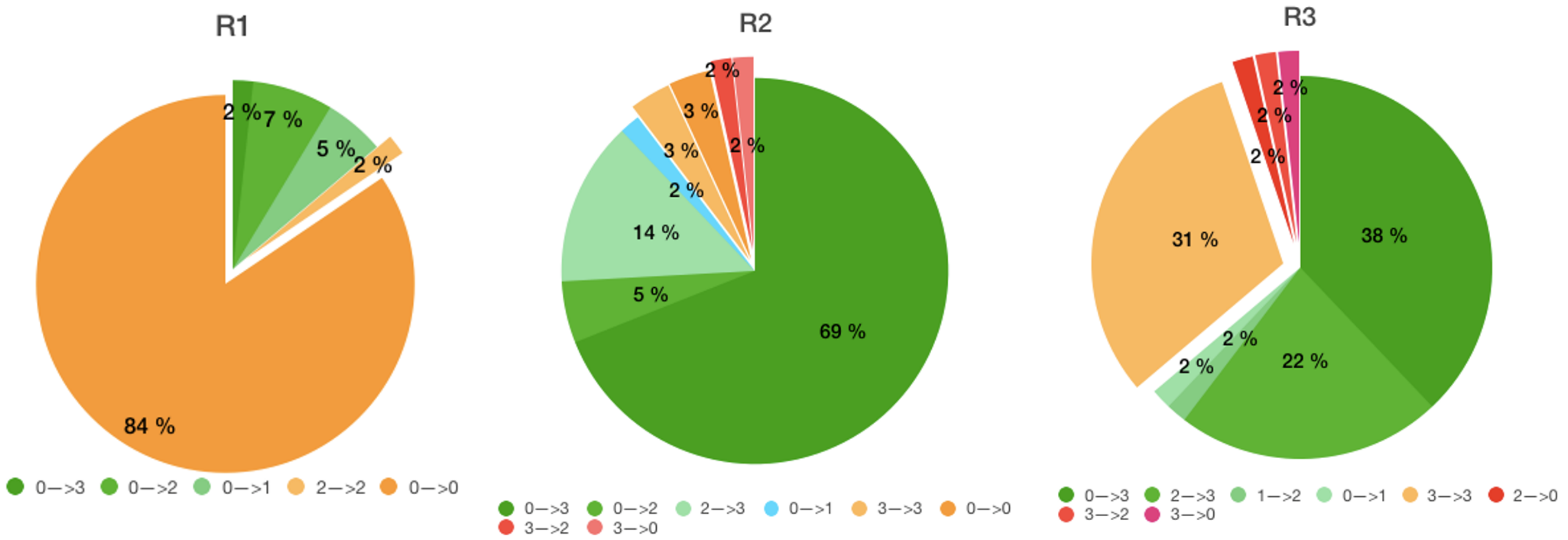

Fig S3: Pie chart of the 3 analysed regions (R1-R3), showing the changes in the image quality after iMAR application (Table 1). The green and blue colours represent improved image quality, the orange colour represents no change, and the red color represents reduced quality. The central tendency differed significantly between the groups (corrected and uncorrected), R1: z=2.57, *p*=0.01, R2: z=6.41, *p*<0.001, R3: z=4.90, *p*<0.001.

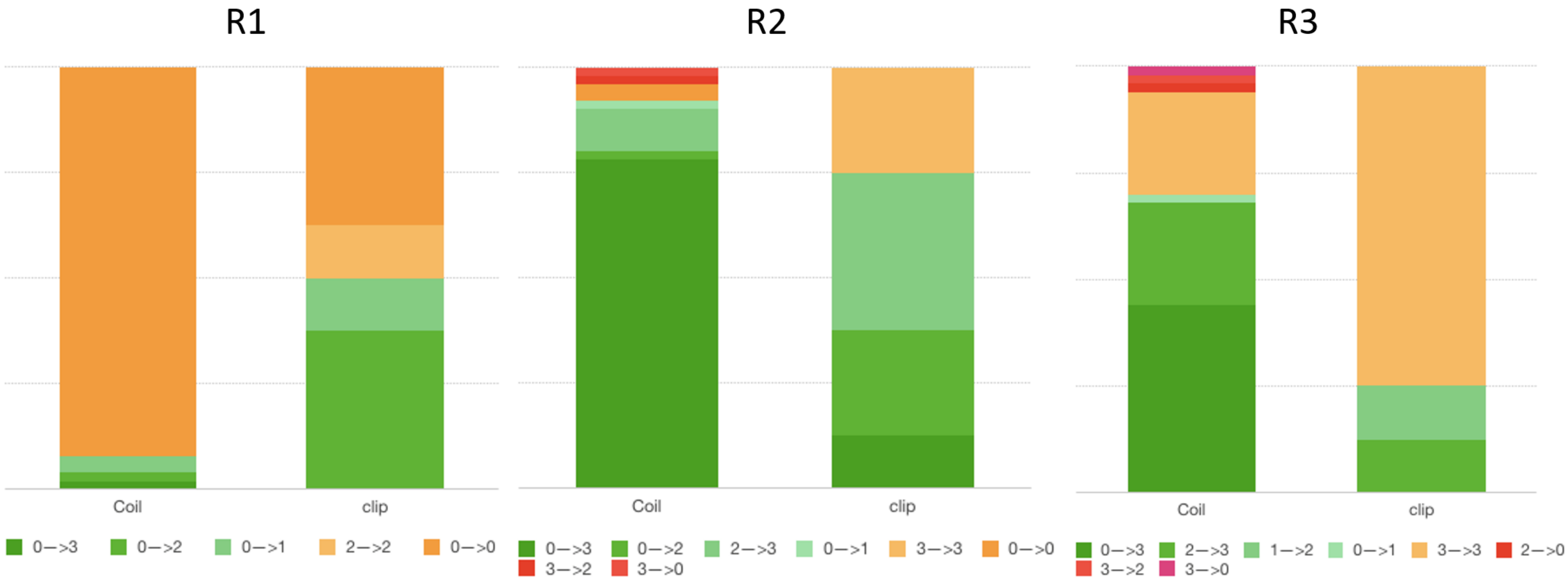

Fig S4: Stacked column chart comparing image quality improvement between patients with coils and those with clips. In R1, the improvement was better in patients with clips. In R2 and R3, the improvement was better in patients with coils.

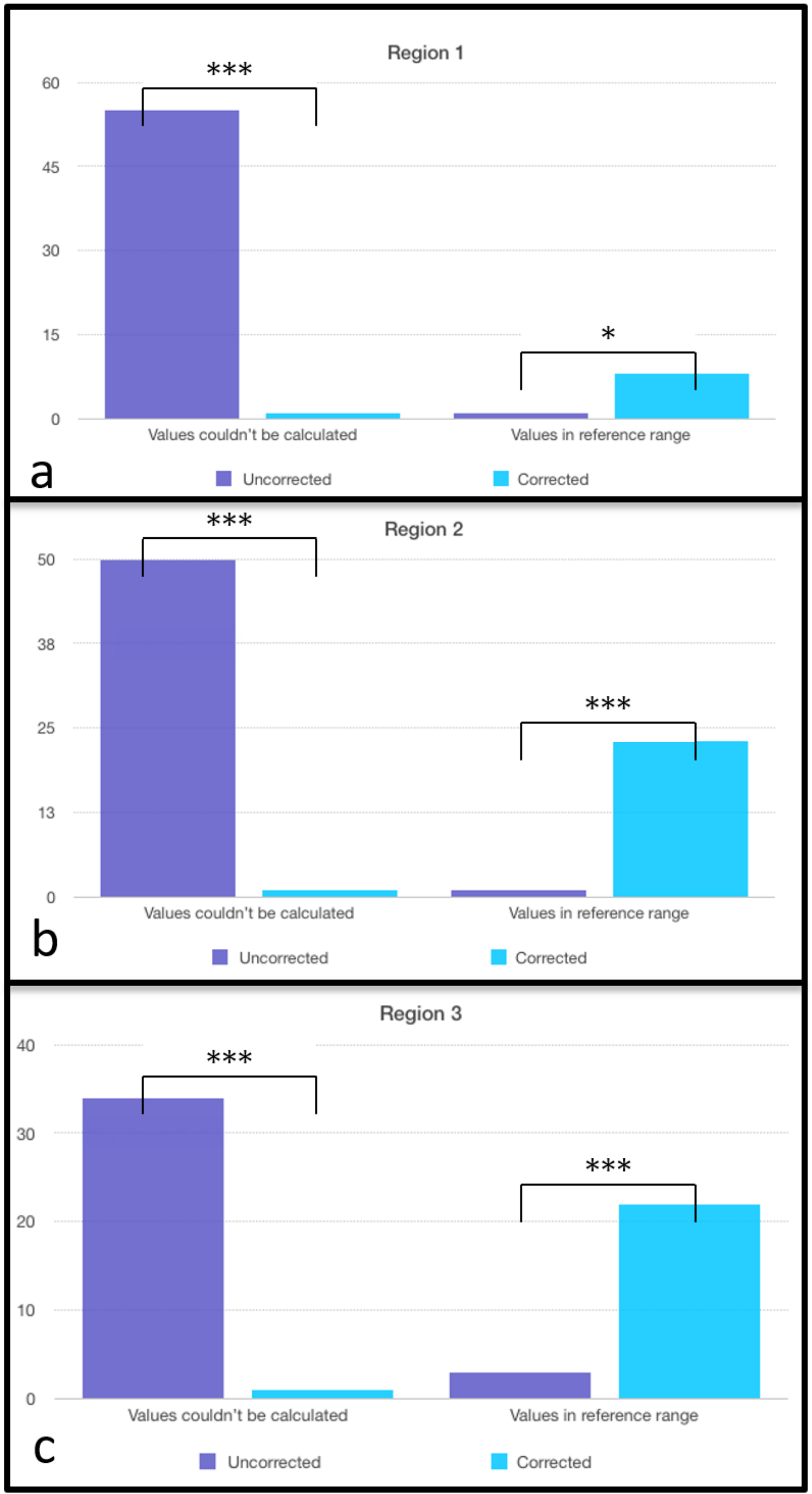

Fig S5: Column charts of the 3 different regions of analysis: a) region 1, b) region 2, and c) region 3, depicting the comparison between the corrected (light blue) and uncorrected (dark blue) datasets in the number (y-axis) of ROIs that could not be calculated due to software error and in the number of ROIs obtained within the reference range. The results of the comparison were statistically significant. * $p < 0.05$, ** $p < 0.01$, *** $p < 0.001$.

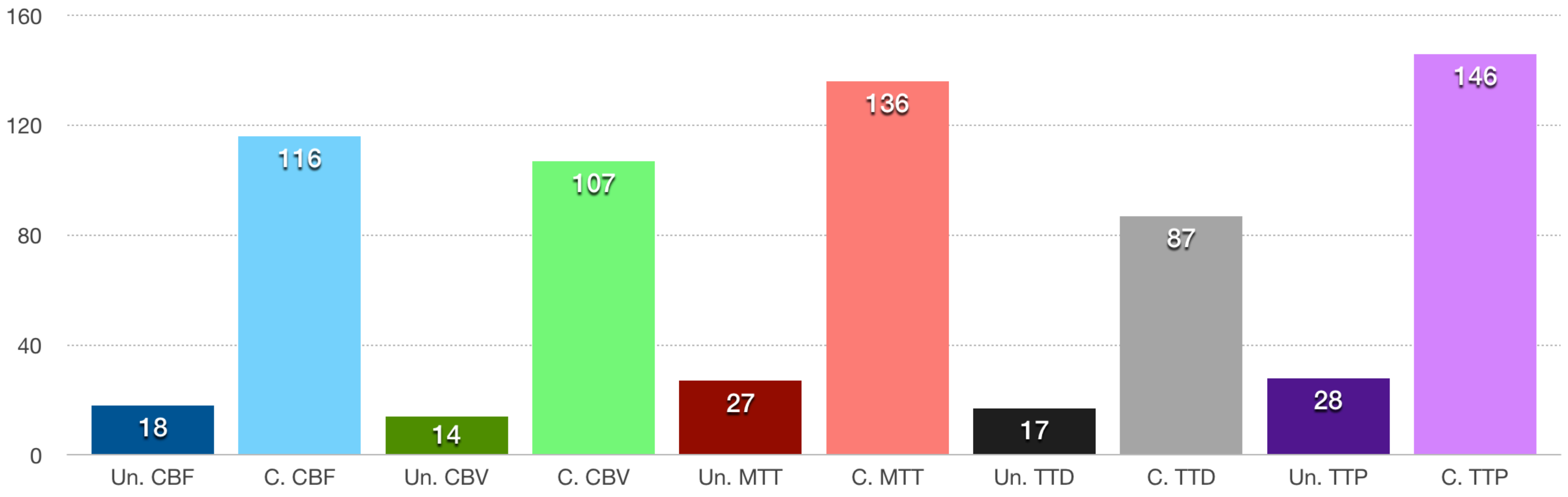

Fig S6: Column bars of the different perfusion parameters obtained from the uncorrected (Un.) and corrected (C.) datasets with the frequency (y-axis) of each parameter within the reference range showing an overall increase in the number of ROIs with perfusion values within the reference range in the corrected dataset, i.e., after the application of iMAR. The bars also show that TTP gave the best results and MTT the second-best. The results of the statistical analysis were not significant in the uncorrected dataset (chi-square = 8.7, $p = 0.07$) but were significant in the corrected dataset (chi-square = 66.62, $p < 0.001$). CBF = cerebral blood flow, CBV = cerebral blood volume, MTT = mean transit time, TTD = time to drain, TTP = time to peak.
